# The Combination of Lymph Node Transfer and Excisional Procedures in Bilateral Lower Extremity Lymphedema: Clinical Outcomes and Quality of Life Assessment with Long-Term Follow-Up

**DOI:** 10.3390/jcm11030570

**Published:** 2022-01-24

**Authors:** Luigi Losco, Alberto Bolletta, Alessandro de Sire, Shih-Heng Chen, Gokhan Sert, Dicle Aksoyler, Jonathan Velazquez-Mujica, Marco Invernizzi, Emanuele Cigna, Hung-Chi Chen

**Affiliations:** 1 Department of Plastic Surgery, China Medical University, Taichung 404, Taiwan; luigi.losco@gmail.com (L.L.); alb.bolletta@gmail.com (A.B.); drgokhansert@gmail.com (G.S.); drdicleaksoyler@yahoo.com (D.A.); drjonathan.vm@gmail.com (J.V.-M.); 2 Department of Translational Research and New Technologies in Medicine and Surgery, University of Pisa, 56126 Pisa, Italy; 3 Physical Medicine and Rehabilitation Unit, Department of Medical and Surgical Sciences, University of Catanzaro “Magna Graecia”, 88100 Catanzaro, Italy; alessandro.desire@unicz.it; 4 Department of Plastic Surgery, Chang Gung Memorial Hospital, Taoyuan City 33305, Taiwan; shihhengche999@hotmail.com; 5 Department of Plastic Reconstructive and Aesthetic Surgery, Ankara Training and Research Hospital, Ankara 06010, Turkey; 6 Division of Plastic and Reconstructive Surgery Nicklaus Children’s Hospital, Miami, FL 33155, USA; 7 Physical and Rehabilitative Medicine, Department of Health Sciences, University of Eastern Piedmont, 28100 Novara, Italy; marco.invernizzi@med.uniupo.it; 8 Translational Medicine, Dipartimento Attività Integrate Ricerca e Innovazione (DAIRI), Azienda Ospedaliera SS. Antonio e Biagio e Cesare Arrigo, 15121 Alessandria, Italy

**Keywords:** lymphedema, lymphedema of the lower limbs, LYMQoL questionnaire, quality of life, vascularized lymph node transfer, lymph node flap, bilateral lymphedema, primary lymphedema, modified Charles procedure, liposuction

## Abstract

Background: Bilateral lower extremity lymphedema is a rare and invalidating condition that poses a great challenge to the scientific community, and deeply affects the quality of life (QoL) of affected patients. A combined protocol consisting of lymph node transfer and a reductive method have never been reported for the treatment of this condition, except for small case series with brief follow-up periods. Methods: This retrospective study analyzed data of 29 patients, mean age 51 ± 17.1 years, who had been diagnosed with bilateral lower extremity lymphedema. Gastroepiploic vascularized lymph node transfer was performed in all the patients, and an excisional procedure was associated according to the clinical stage. Clinical history, circumferential limb measurements, complications, episodes of cellulitis, and responses to the Lymphedema Quality of Life Questionnaire were analyzed. Results: The mean follow-up was 38.4 ± 11.8 months. A significant reduction in the episodes of cellulitis per year was observed (*p* < 0.001). In our series, BMI and duration of symptoms were significantly related to the development of cellulitis during the postoperative period, *p* = 0.006 and *p* = 0.020, respectively. The LYMQoL questionnaire showed a significant quality of life improvement from 3.4 ± 0.9 to 6.2 ± 0.8 (*p* < 0.05). Conclusions: An integrated approach is essential for the treatment of bilateral lower extremity lymphedema: reductive and reconstructive methods are complementary to achieve a successful outcome. Timely treatment and BMI reduction are relevant in order to decrease the number of episodes of cellulitis. An attentive follow-up is necessary to identify recurrence and treat affected patients in time.

## 1. Introduction

Bilateral lower extremity lymphedema is a major source of morbidity. It is a rare condition that poses a great challenge to the scientific community, and deeply affects the quality of life (QoL) of affected patients [1,2,3]. Lymphedema is usually not life threatening, but because of the swelling of the affected limb, discomfort, pain, loss of function, and recurrent infections may occur. Moreover, it has a very deleterious effect on self-esteem, and causes impairments in body perception [4,5].

Primary lymphedema is the result of a progressive swelling without relation to any underlying medical condition; it is caused by abnormal development of lymphatic system whose onset ranges from childhood to adult age, and it could show greater severity in cases of early onset [6]. In secondary cases, the impairment of the lymphatic system could be caused by direct trauma, infection, surgery for cancer, or radiotherapy. It leads to a disruption of the lymphatic channels and a subsequent compromised flow of lymph from the limb; it is a major cause of distress for patients and frustration for physicians. In secondary cases, patients develop edema due to a medical condition, and they are attended to with a closer follow-up; in primary cases, instead, the diagnosis may be delayed [7,8,9,10]. Currently, there is no cure for this condition, and the therapeutic goal is to reduce progression of the disease and secondary complications; early diagnosis is important in order to set an effective physical therapy protocol and treatment [11,12,13,14,15].

Surgical procedures for lymphedema are generally divided in two classes: reductive methods, such as extensive therapeutic lipectomy or direct excision, such as Charles’ procedure or radical reduction with preservation of perforators (RRPP); the second category is composed of physiologic methods, such as vascularized lymph node transfer (VLNT) and lymphaticovenular anastomosis (LVA).

In our experience, bilateral lower extremity lymphedema can be tough and challenging. Mild cases can be underdiagnosed because the patient cannot compare the affected limb with the healthy one, and may easily neglect this progressive disease; moreover, concomitant obesity can conceal mild lymphedema. In advanced conditions, a severe impairment in function and quality of life can be complained of, even if a successful bilateral lower limb surgery is performed. In the current literature, few studies have reported surgical outcomes and algorithms for the treatment of bilateral lower limb lymphedema; furthermore, they have described small samples or even case reports, neglecting the approach to the advanced stages of the condition [16,17].

The aim of this retrospective cohort study on bilateral lower extremity lymphedema was to describe our combined approach and surgical tips, and to investigate patients’ outcomes and quality of life with a conspicuous follow-up period. Even if bilateral lymphedema seems to simply be the lymphedema of both limbs, indeed, the problems are more than doubled.

## 2. Materials and Methods

Patients affected with lymphedema and treated from January 2011 to June 2019 at China Medical University Hospital were retrospectively reviewed. Inclusion criteria of the study were bilateral lower limb lymphedema, progressive swelling not respondent to decongestive therapy, and treatment with our combined surgical approach, with at least 24 months of continuous follow-up. Patients undergoing primary non-synchronous treatment for the two limbs were excluded from this study.

Twenty-nine patients were diagnosed with bilateral lymphedema and were included in the present study. The diagnosis was based on medical records, clinical examination, and lymphoscintigraphy. Patients were classified as stage II or III lower limb lymphedema, as defined by the International Society of Lymphology (ISL) [18].

Twenty patients were affected with secondary lymphedema: the underlying pathology was endometrial cancer in six patients, ovarian cancer in nine patients, and prostate cancer in five patients. Nine patients did not show other medical conditions that could be linked to the development of lymphedema: they were diagnosed with primary lymphedema according to the past medical history. The patients underwent circumferential limb and body mass index measurements; duration of symptoms and the number of preoperative and postoperative episodes of cellulitis were also documented, along with complication and re-operation rate. Antibiotics were administered if lymphedema was complicated with infection.

Circumferential measurements were taken at four levels: midfoot, ankle (between medial and lateral malleoli), mid-calf, and mid-thigh. All the patients underwent a gastroepiploic (GE)-VLNT, and it was combined with either an extensive liposuction or a major reductive procedure (Charles’ or RRPP procedure), according to the clinical grade. See Table 1 for patient details. The Lymphedema Quality of Life (LYMQoL) Questionnaire [19] was administered preoperatively, and 12 and 24 months after surgery. The questionnaire provided questions based on patients’ function, appearance, symptoms, and mood. The greater the overall score (from 1 to 10) the better the quality of life was for the patient.

### 2.1. Surgical Techniques

**Figure 1:**
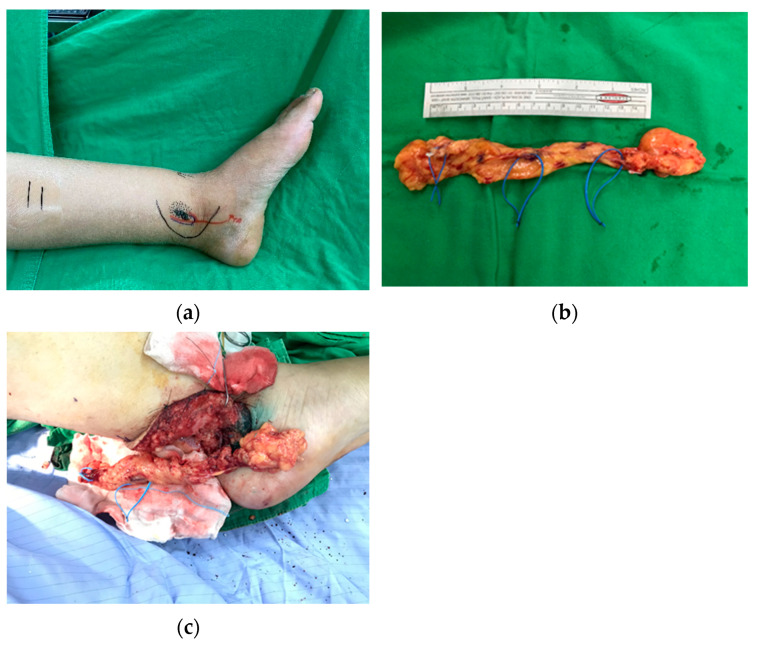
(**a**) Preoperative picture. The curved incision line is depicted in black. The course of posterior tibial artery (PTA) is highlighted, and the future position of gastroepiploic lymph node flap is simulated. (**b**) Gastroepiploic lymph node flap. The flap is positioned on a separate table for vessel preparation under the operative microscope. The vessel loops trace the path of the gastroepiploic vessels. (**c**) After the whole flap is anastomosed to one ankle, the vessels highlighted by the median loop are divided and the distal half of the flap is transferred to the contralateral ankle.

All patients underwent a combined procedure. A GE-VLNF transfer was combined with a different excisional procedure, according to the patient’s clinical grade. The GE-VLNF was harvested by a laparoscopic surgeon, as previously described [20]. All of the flaps were divided into two segments and transferred to both extremities at a the medial inframalleolar area (posterior tibial vessels) (Figure 1a–c). If a primary closure was not possible, a split-thickness skin graft was used to cover the flap in order to prevent external compression of the pedicle.

**Figure 2:**
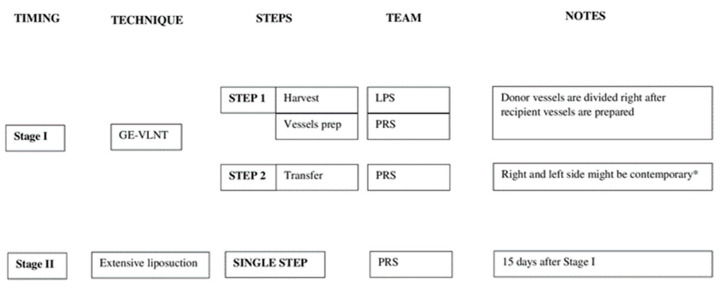
Authors’ two-stage protocol for bilateral lymphedema treatment—moderate clinical grade. * Two microscopes should be available. GE-VLNT—gastroepiploic-vascularized lymph node transfer; LPS—laparoscopic team; PRS: plastic reconstructive surgery team.

**Figure 3:**
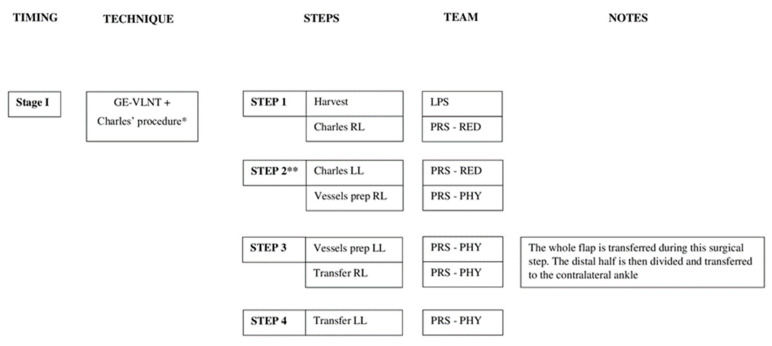
Authors single-stage protocol for bilateral lymphedema treatment—advanced clinical grade. GE-VLNT—gastroepiploic-vascularized lymph node transfer; LPS—laparoscopic team; PRS-RED—reduction team; PRS-PHY—physiologic procedure team; RL—right lower limb; LL—left lower limb. * RRPP could be performed instead of Charles’ procedure. ** Tourniquet is released before vessel preparation.

In 22 cases, a 2-stage approach was performed. Two weeks after bilateral GE-VLNT, an extensive therapeutic lipectomy with Brorson’s technique was carried out to reduce the lymphatic burden of the lower limbs [21]. Compression garments were applied immediately after the surgery. In seven cases, RRPP or modified Charles’ procedure and VLNT were performed in a single stage; in those cases, teamwork is necessary in order to complete a double reductive procedure with subsequent double lymph node transfer in reasonable operative time (we recommend building a reductive and a reconstructive team in order to avoid surgeon fatigue). The reductive techniques were described in our previous reports [22,23]. For the sake of clarity, a scheme was used to describe the surgical protocol used for each clinical grade (Figure 2 and Figure 3).

### 2.2. Statistical Analysis

Statistical analysis was performed using SPSS Statistics software package version 25 (IBM Corp. SPSS Statistics for Windows, Armonk, NY, USA). Parametric data were provided as mean ± standard deviation and range. Wilcoxon signed ranks test was used for preoperative and postoperative comparison of episodes of cellulitis/year and Quality of Life. The influence of duration of symptoms and BMI on the incidence of postoperative cellulitis was analyzed by the two-tail Mann–Whitney test. The significance was set at a value of *p* < 0.05.

## 3. Results

Patients were first evaluated at a mean age of 51 ± 17.1 years, and the mean BMI was 25.5 ± 2.8 kg/m^2^. The patients had reported swelling or multiple symptoms for a mean duration of 32.5 ± 24.7 months, and they presented an average circumference reduction of 4.2 ± 3.3 cm. Before surgery, the patients presented with 2.7 ± 0.8 episodes of cellulitis per year. In the mean follow-up time, the patients reported 0.7 ± 0.8 episodes of cellulitis per year. The improvement was significant (*p* < 0.001). In our series, higher preoperative BMI was significantly related to the development of postoperative cellulitis, *p* = 0.006. Development of postoperative cellulitis was also significantly associated with the duration of symptoms, *p =* 0.020. (Table 1 and Table 2)

All transferred lymph nodes survived. We did not observe any major complications. We reported 31% of minor complications. Throughout the follow-up period, we did not experience any donor site morbidity. The average follow-up of the patients was 38.4 ± 11.8 months. The most frequent complication was hypertrophic scarring (17%): 5 patients were treated with steroid injections, and lately underwent excision of crypts (deep depressions in the skin-grafted area); skin grafting was repeated. One patient complained of persistent numbness of the right leg. There was 1 patient who presented with persistent bilateral erythema and recurrent infections 1 year after treatment; he was severely obese (BMI 36.5 kg/m^2^) and had been affected by primary lymphedema for 13 years. One patient experienced partial skin graft loss that required re-grafting. Recurrence of infection was seen in three limbs, and it was controlled with antibiotics. Furthermore, no ulcerations were noted during the follow-up period, except in one patient, who experienced severe infection of the toe, and toe amputation was deemed necessary.

**Figure 4:**
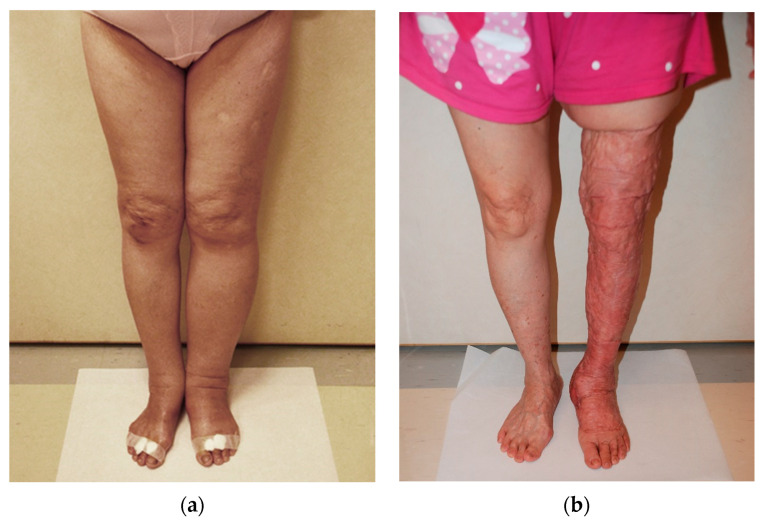
(**a**) Preoperative picture of a 59-year-old female patient. The patient was affected with stage II bilateral secondary lymphedema; the left lower limb was more severely affected. Bilateral GE-VLNF was transferred, and suction lipectomy was performed during the second surgical stage. A unilateral lymphedema progression was observed during follow-up, and we had to plan a step forward in the surgical protocol: a Charles’ procedure was performed to treat the left lower limb. (**b**) Twenty-four-month post operative picture shows a satisfactory result. Charles’ procedure was carried out on the left limb. Right limb did not require any further treatment.

In three cases, a recurrence of the symptoms after VLNT and liposuction was observed. In two cases, it was a bilateral recurrence, and in one case it was unilateral (Figure 4). A modified Charles’ procedure was performed for those five limbs (Table 3).

The LYMQoL questionnaire was administered 24 months postoperatively; the overall score significantly improved from 3.4 ± 0.9 to 6.2 ± 0.8 (*p* < 0.05). Function, appearance, symptoms, and mood sections of the questionnaire led to a significant QoL improvements (all *p* < 0.05). A stratification, according to surgical protocol, was performed to avoid any bias in QoL evaluation: Group A: VLNT and liposuction; Group B: VLNT and Charles/RRPP (Group B also included the three re-operated patients) (Table 4).

## 4. Discussion

To the best of our knowledge, this is the largest account on a bilateral lower limb lymphedema cohort managed in a single center; the patients were treated with our integrated approach and showed improvement of both clinical conditions and quality of life.

Combination of physiologic and excisional methods was reported to be effective in the treatment of moderate and advanced stages unilateral lymphedema; however, patients with bilateral lymphedema were always excluded during study design in order to allow a regular assessment of the reduction in affected limb size as compared with the healthy one [24,25,26,27]. The safety and efficacy of a combined protocol had never been reported for bilateral lower extremity lymphedema, except for a small case series regarding four patients with a short follow-up [17].

Vascularized lymph node transfer is a safe and effective technique; it significantly reduces volume and circumference of the limb, and restores immunocompetence of the distal extremity [20,28]. Many VLNF options are available. Since all VLNTs reported in the literature achieved acceptable outcomes, we decided to exploit the GE-VLNT, based on the satisfactory clinical outcomes, low donor site morbidity, and rapid recovery (due to laparoscopic harvest) [29,30]. The GE-VLNF is particularly suited to our purpose because it can be divided in two halves for a bilateral inset; furthermore, we do not have to exploit a second donor site.

Thorough comprehension of the physiopathology of lymphedema is preeminent to plan an effective treatment protocol. If lymph stagnates, a pro-inflammatory environment leads to adipogenesis and to an excess of subcutaneous adipose tissue [31]. As the disease progresses, the inflammatory cytokines induce fibroblasts recruitment, collagen deposition, and consequent fibrosis. In a later stage, the skin also becomes affected: the rough surface and the local impairment of the immune system make proper hygiene difficult and cause recurrent infections [32]. Thus, the principal target is to lower the volume of the limb while restoring lymphatic drainage, with the aim of preventing infections. The efficacy and the safety of lipectomy in patients affected with lymphedema had been already evaluated: it should not be considered just an excisional procedure [21,24]. The benefits are both mechanical and cellular-/humoral-mediated: (1) reduction in the lymphatic burden; (2) removal of excess subcutaneous tissue to indirectly improve lymphatic drainage; (3) anti-inflammatory effect due to the removal of proinflammatory cells and cytokines; (4) reduction or stop of the downfall of lymphatic vessels; (5) lessening of the infectious episodes; (6) increased blood flow to the skin [33,34].

In advanced cases, the fibrotic component of the subcutaneous tissue is too hard for removal by suction lipectomy, and major excisional procedures, such as RRPP or modified Charles’ procedure, are necessary to dramatically reduce the lymphatic load. The combination with transferred lymph node transfer helps to improve the condition of the entire limb, especially at the most distal part (foot and toes) where the tissues cannot be removed circumferentially as in the thigh and leg [35,36,37].

The timing of a combined surgery is really important, especially in bilateral extensive surgeries. During the combined modified Charles’ procedure with GE-VLNT, if possible, we prefer to work divided into three teams alternating in multiple well-defined steps. After the VLNF is harvested laparoscopically, one team takes care of the reductive procedure, and another team performs the microsurgical steps in order to reduce the operative time and the surgeon’s fatigue. The surgical steps are described in Figure 3. In case only one microscope is available, we recommend first performing anastomoses of the entire flap to one ankle with the aim to interrupt the ischemia time. After the recipient vessels of the contralateral ankle are prepared, the distal half of the flap is divided and transferred (Figure 1c).

A dramatic change in cellulitis to 0.7 ± 0.8 episodes of cellulitis per year (*p* < 0.001) was reported. One patient presented with persistent bilateral erythema and recurrent infections one year after treatment. He had been suffering from primary lymphedema for 13 years and was severely obese (BMI 36.5 kg/m^2^). Obesity is considered a risk factor for the development of lymphedema [38] and has already been investigated as predictor of unfavorable postoperative outcomes in various clinical settings [39,40]. We established a significant relation between higher BMI and postoperative cellulitis, *p* = 0.006.

It was reported that duration of symptoms is proportional to severity of lymphedema due to an earlier tissue fibrosis [41]; our findings are consistent with these studies, and we established a significant relation between the duration of symptoms and the occurrence of postoperative cellulitis, *p* = 0.020.

In advanced lymphedema, both preoperative and postoperative closer follow-up is paramount in order to avoid worsening of the clinical grade; on the other hand, patients’ compliance through postoperative skin care (especially of the foot) and physical rehabilitation are important. Patients must nourish the skin graft with moisturizing cream, and prevent formation of any hypertrophic scar by wearing compression garments. Intralesional steroid injection for hypertrophic scars may be necessary [22].

In 3 cases (10%), we observed recurrence of symptoms and lymphedema progression after VLNT followed by extensive liposuction. One patient (secondary lymphedema) showed unilateral progression of lymphedema during follow-up; in two cases (primary lymphedema), a bilateral progression of symptoms was reported. We had to plan a step forward in the surgical protocol for these patients, and a modified Charles’ procedure was performed.

In refractory cases, we are more liberal in offering the modified Charles’ procedure because we had already provided our reconstructive method of choice and we have to firmly avoid any further progression of the disease/fibrosis that can lead to more severe issues, such as regional immune compromise, trophic change of the foot and toes, overcrowding, deformities of toes, and finally toe amputation [26,32,42]. In our series, operations for refractory lymphedema were performed in 1/20 cases (5%) after secondary lymphedema and 2/9 cases (22%) after primary lymphedema. A proportion of 4 out of 5 limbs (80%) showed a primary etiology; these data are consistent with Cheng et al. [41]. They reported that primary cases show a greater severity, and this could be due to the earlier age of onset and the consequent long-lasting lymphedema causing associated fibrotic changes.

To the best of our knowledge, refractory lymphedema has never been reported following VLNT, while it has been described after LVA [43]. An attentive follow-up is necessary to identify the recurrence and treat those patients in time.

Double level inset into the middle and distal aspect of the limb provides uniform size improvement along the entire extremity; in our institution, it is the standard of care for patients affected with unilateral lymphedema [20,24,26]. A single level inset was reported to be safe and effective in the treatment of bilateral lymphedema, even only with a short-term follow-up [17,35]. In our series, the relapsing cases were affected with late-stage II lymphedema; we could assume that single level VLNT was not sufficient due to the severity of the condition.

Nowadays, there is a growing interest in the assessment of QoL following reconstructive surgery [44,45,46]. Recent literature about unilateral lower extremity lymphedema reported very good results in terms of QoL with a 2–3-fold increase [35,41]. The authors used the same questionnaire that we used; however, the affected cohorts and treatment protocols were non-homogeneous: any comparison could be biased. Besides which, in our study, the reported QoL improvement was lower than theirs. We think that dealing with 29 patients affected with bilateral lymphedema is not the same as dealing with a cohort affected by unilateral lymphedema. The QoL of our patients was deeply affected: in everyday life they did not have the relief of a healthy limb, and everything from wearing clothes to undergoing surgery was more complicated. It is clear to us that a patient with both affected limbs should have a different QoL assessment than a unilateral cohort, and that mixed cohorts (unilateral and bilateral patients) should not undergo a quality of life evaluation during the same study.

In our series, the QoL improved significantly in function, appearance, symptoms, and mood domains: the infection was controlled, and the motility and ultimately the confidence were increased. The overall satisfaction, assessed by the LYMQoL, increased significantly from 3.4 ± 0.9 to 6.2 ± 0.8 on a scale of 0 to 10. To avoid any bias in QoL evaluation, a stratification according to surgical protocol was performed (Group A and Group B) (Table 4).

In group B, function and symptoms scores mostly contributed to the QoL improvement; although, after the skin graft, the range of movements of the leg was decreased temporarily. The motility of the patients markedly improved as they were relieved from the heaviness and the morbidity of the affected leg; furthermore, they could easily find suitable clothes. We think that mood did not dramatically enhance because patients of group B (mostly affected with primary lymphedema) with longer duration of symptoms were largely discouraged and showed a subtle or evident depression. In group A, appearance and mood domains mostly contributed to the QoL gain: patients were grateful because they got an easier life, and the cellulitis was downgraded to almost zero. The appearance domain markedly improved because they gained novel self-confidence and social life.

In both groups, the QoL enhanced significantly. Group A showed a greater satisfaction with an average postoperative QoL 6.3 ± 0.8 (group B, 6 ± 0.8); moreover, the preoperative QoL of Group A was also greater, as we could expect. The questionnaire showed a greater mean improvement in Group B (3.4 ± 0.5) than group A (2.6 ± 0.5); these data tell us that the most severely affected and aggressively treated group showed a larger improvement. We might think that the quality of life in patients affected with bilateral lymphedema could not be investigated with the LYMQoL Questionnaire, because it was designed for unilateral lymphedema. Or maybe, these facts are telling us that the way of thinking of the physician should be changed.

The present study has some limitations, being both retrospective and monocentric. The bilateral lymphedema population is small, even though it is the largest compared with the previous literature. Anyway, the surgical management, postoperative outcomes, and QoL of a tough population were detailed in this study; moreover, we attempted to offer a precise, surgical step-based protocol with a long-term follow-up—these are the strengths of this study.

## 5. Conclusions

An integrated approach is essential for the treatment of bilateral lower extremity lymphedema: reductive and reconstructive methods are complementary in achieving a successful outcome. Timely treatment and reduction in BMI are important to powering down the postoperative cellulitis rate. The most severely affected group reported the greatest quality of life improvement, even if those patients underwent a more invasive surgery and reported more complications. An attentive follow-up is necessary to identify recurrence and to treat those affected patients in time.

## Figures and Tables

**Table 1:** Patients and outcomes.

Variable	Value (Rate)
Patients	29
Age—years	
Mean ± SD	51 ± 17.1
Range	15–75
Gender	
Female	18 (62%)
BMI—kg/m^2^	
Mean ± SD	25.5 ± 2.8
Range	22–36.5
Etiology	
Primary	9 (31%)
Secondary	20 (69%)
Duration of symptoms—months	
Mean ± SD	32.5 ± 24.7
Range	20–158
Circumference improvement—cm	
Mean ± SD	4.2 ± 3.3
mid-thigh	5.1 ± 1.1
mid-calf	6.9 ± 5
ankle	2.9 ± 0.7
mid-foot	1.7 ± 0.5
Follow-up—months	38.4 ± 11.8

**Table 2:** Statistical Analysis—factors affecting postoperative cellulitis development.

	Postoperative Cellulitis	No Postoperative Cellulitis	*p*-Value
Duration of symptoms (months)	39.1 ± 34.2	26.3 ± 4.9	0.020
Body mass index (kg/m^2^)	26.5 ± 3.4	24.3 ± 1.4	0.006

**Table 3:** Complications and re-operations.

Complications	Value (Rate)
Overall complications	9 (31%)
Recurrent infections	3 (10%)
Partial skin graft loss	1 (3%)
Persistent erythema	1 (3%)
Persistent numbness (unilateral)	1 (3%)
Hypertrophic scarring	5 (17%)
Revisional surgery	
Skin grafting	6 (20%)
Crypt excision	5 (17%)
Toe amputation	1 (3%)
Re-Operations *	3 (10%)

* Due to recurrence of symptoms after VLNT and liposuction.

**Table 4:** Quality of life assessment—Lymphedema Quality of Life (LYMQoL) Questionnaire.

Features	Preoperative LYMQoL	Postoperative LYMQoL	Improvement	*p*-Value
Group A * (overall)	3.7 ± 0.6	6.3 ± 0.8	2.6 ± 0.5	<0.001
Function	28.2 ± 1.1	24.2 ± 1.3	4 ± 0.7	
Appearance	25.3 ± 1	18.9 ± 1.5	6.3 ±1.3	
Symptoms	15.8 ± 1.2	12.1 ± 1.3	3.7 ± 0.9	
Mood	19.3 ± 1	11.7 ± 1.4	7.5 ± 1.3	
Group B * (overall)	2.6 ± 1	6 ± 0.8	3.4 ± 0.5	<0.001
Function	30.6 ± 1.2	24 ± 2.4	6.6 ± 1.8	
Appearance	26.9 ± 1.3	21.6 ± 2.8	5.3 ± 2	
Symptoms	18.3 ± 1.1	12.2 ± 2.6	6.1 ± 1.9	
Mood	22.7 ± 1.1	18.3 ± 2.6	4.4 ± 1.9	
Total (overall)	3.4 ± 0.9	6.2 ± 0.8	2.8 ± 0.6	<0.001

* Group A: VLNT and liposuction; Group B: VLNT and Charles/RRPP and re-operated patients. LYMQoL values. Overall score (1–10 points). The four domains (the lower the score, the better the quality of life): Function (32 points); Appearance (28 points); Symptoms (20 points); Mood (24 points).

## Data Availability

The data presented in this study are available on request from the corresponding author H.-C.C. The data are not publicly available due to privacy restrictions.
